# DNA Damage Response Regulation by Histone Ubiquitination

**DOI:** 10.3390/ijms23158187

**Published:** 2022-07-25

**Authors:** Miyu Sekiguchi, Nobuko Matsushita

**Affiliations:** Laboratory of Hygiene and Public Health, Department of Medical Technology, School of Life and Environmental Science, Azabu University, 1-17-71 Fuchinobe, Chuo-ku, Sagamihara 252-5201, Japan; ql2108@azabu-u.ac.jp

**Keywords:** ubiquitination, chromatin, DNA repair, histone modifications

## Abstract

Cells are constantly exposed to numerous genotoxic stresses that induce DNA damage. DNA double-strand breaks (DSBs) are among the most serious damages and should be systematically repaired to preserve genomic integrity. The efficiency of repair is closely associated with chromatin structure, which is regulated by posttranslational modifications of histones, including ubiquitination. Recent evidence shows crosstalk between histone ubiquitination and DNA damage responses, suggesting an integrated model for the systematic regulation of DNA repair. There are two major pathways for DSB repair, viz., nonhomologous end joining and homologous recombination, and the choice of the pathway is partially controlled by posttranslational modifications of histones, including ubiquitination. Histone ubiquitination changes chromatin structure in the vicinity of DSBs and serves as a platform to select and recruit repair proteins; the removal of these modifications by deubiquitinating enzymes suppresses the recruitment of repair proteins and promotes the convergence of repair reactions. This article provides a comprehensive overview of the DNA damage response regulated by histone ubiquitination in response to DSBs.

## 1. Introduction

Many types of DNA damage occur in cells, of which the most serious is DNA double-strand break (DSB) caused by ionizing radiation, anticancer chemotherapeutic drugs, and endogenous replication fork collapse [1]. Anticancer drugs include cross-linking agents, such as cisplatin and mitomycin C, alkylating agents, such as methyl methanosulfate, and radiomimetic agents, such as bleomycin [2]. Ionizing radiation produces DNA single-strand breaks (SSBs) using water radiolysis-generated radicals [2,3]. Closely opposed SSBs created in complementary DNA strands within a single helical turn lead to DSBs [4]. Since unrepaired DSBs result in the accumulation of chromosomal aberrations and translocations, their repair is essential for cell survival [5]. Two major pathways are involved in DSB repair, viz., nonhomologous end joining (NHEJ) and homologous recombination (HR) [6]. NHEJ can occur throughout the cell cycle and rejoin break ends without sequence homology. It is generally considered to be error-prone, owing to its minimal end processing [7,8]. Contrarily, HR is activated in the S and G2 phases of the cell cycle and when sister chromatids can be used as templates for repair [9]. HR is initiated by the 5′ DNA end resection and the binding of Rad51 to single-stranded DNA. The later stages of HR involve the search for a homologous sequence, formation of a displacement loop (D-loop) by single-stranded DNA invasion, removal of Rad51, and repair synthesis to copy the donor template to restore the genetic information at the break sites [10].

When DSBs occur, the early response is primarily conducted using ataxia-telangiectasia mutated (ATM) protein kinase [11,12]. At DSB sites, ATM phosphorylates many substrates and induces complex downstream posttranslational modifications (PTMs) to promote DNA repair [12,13,14]. Signaling pathways in early responses to DSBs promote the recruitment of 53BP1 and BRCA1 in later responses [8]. Accumulated ATM phosphorylates H2AX, but DNA-dependent protein kinase catalytic subunit (DNA-PKcs), ataxia telangiectasia, and Rad3-related (ATR) also phosphorylate H2AX [15,16,17]. Additionally, increased levels of γH2AX on either side of the DSB further promote chromatin decondensation and DSB repair [18]. Chromatin structure is a key determinant of DSB repair pathway choice. The functional unit of chromatin is the nucleosome, which generally contains 146 base pairs of DNA wrapped around a histone octamer containing four types of histone proteins, viz., H2A, H2B, H3, and H4 [19,20]. Histone proteins have an unstructured amino terminus or C-terminus that extends out from the nucleosome and is known as the histone tail. The histone tail region is enriched in basic amino acids, such as positively charged lysine and arginine that interact with the negatively charged phosphate groups of DNA [21]. The histone tail is subjected to various posttranslational modifications, such as acetylation, methylation, phosphorylation, and ubiquitination. These modifications affect the interaction of histone proteins with DNA or reader proteins. Therefore, histone modifications play a critical role in the signaling network of the DNA damage repair response [22,23]. Upon DNA damage, various posttranslational modifications (methylation, ubiquitination, acetylation, etc.) of histones form the basis for the recruitment of downstream effectors, resulting in chromatin remodeling and regulating enzymatic signaling cascades [24]. This histone signaling network is organized into multiprotein complexes containing modules that are writers, readers, and erasers of chromatin marks, respectively. The modularity of these complexes integrates different histone modifications and allows for highly complex signaling [24].

Histone ubiquitination alters the chromatin structure before and after repair, propagates damage signals, and serves as a platform for the recruitment of repair proteins, which have been extensively reviewed [8,25,26,27,28]. Ubiquitin is a small protein of 76 amino acids with a C-terminal diglycine tail and is highly evolutionarily conserved across species. Covalent attachment of ubiquitin to an ε-amino group of a lysine residue on the substrate proteins, ubiquitination, is catalyzed by E1-activating enzymes, E2-conjugating enzymes, and E3 ubiquitin ligases. The C-terminal carboxyl group of ubiquitin is first activated by E1 in an ATP-dependent process and enters into a thioester linkage with the catalytic cysteine of E1. Then, ubiquitin is transferred to the catalytic cysteine of E2 through a transesterification reaction. Subsequently, E3 ligase is involved in the transfer of the ubiquitin from E2 to the ε-amino group of a substrate lysine, promoting the formation of an isopeptide bond between the C-terminal glycine residue of ubiquitin and lysine residue. The addition of a single ubiquitin molecule to a target protein is termed monoubiquitination, and monoubiquitination to additional lysine residues of the substrate is termed multiubiquitination. Furthermore, ubiquitin itself has seven lysine residues (lysine 6 (K6), K11, K27, K29, K33, K48, and K63) and an N-terminal amine, through which ubiquitin is attached, forming a polyubiquitin chain termed polyubiquitination. Conversely, deubiquitinating enzymes (DUBs) remove ubiquitin modifications from substrate proteins [29].

Ubiquitination of histones H2A and H2B is highly site-selective and is one of the key posttranslational modifications that regulate DNA damage response. RNF168, RING1A/RING1B in the polycomb repressive complex 1 (PRC1), and BRCA1-BARD1 modify H2AK13/K15, K118/119, and K127/129, respectively. Ubiquitination of these different sites on H2A triggers different physiological responses [25]. In the DNA damage response, H2A ubiquitination by RNF168 regulates HR and NHEJ [30,31,32,33,34], and H2A ubiquitination by BRCA1/BARD1 promotes HR [6,35,36]. Ubiquitination of H2A by the PRC1 complex plays the global function of transcriptional repression and may play the same role around the DNA damage response [37]. The RNF20/RNF40 heterodimer specifically ubiquitinates K120 of H2B and functions in the activation of damage checkpoints and repair initiation [25,36].

These specific ubiquitinations of histones form a crucial component of the regulatory network facilitating the DNA damage response, and recent advances have begun to elucidate the molecular basis of the specificity and mechanisms involved in repair pathway selection. In this review, we focus on the role of histone ubiquitination in the DNA damage response and maintenance of genome stability through the integration of multiple signaling entities and selection of repair pathways.

## 2. Histone Ubiquitination Regulates DNA Repair

### 2.1. H2AK13/15 Ubiquitination by RNF8-RNF168 and Deubiquitination

**Figure 1:**
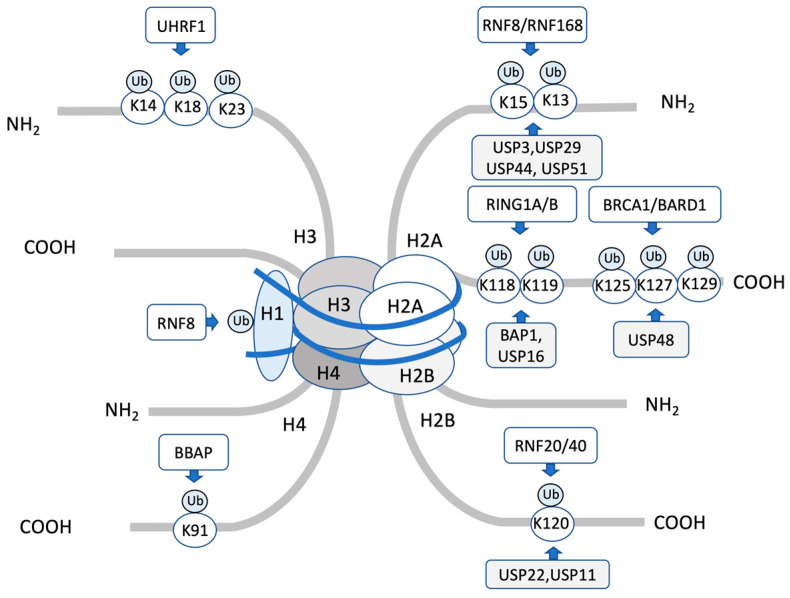
Summary of histone lysine ubiquitination and deubiquitination enzymes involved in DNA damage response. The E3 enzymes are listed above the ubiquitination site, whereas the deubiquitinating enzymes are listed below.

Histone ubiquitination by multiple E3 ligases contributes to appropriate DSB repair, and deubiquitination by multiple DUBs regulates these reactions (Table 1). There are also several proteins with ubiquitin-binding domains, which are the reader proteins that recognize multiple histone ubiquitination (Table 1). RNF168, an E3 ubiquitin ligase, consists of a RING finger domain and two ubiquitin interaction motifs, termed MIU for motif interacting with ubiquitin, that selectively bind to ubiquitin chains. The RING finger domain of RNF168 is critical for its ubiquitin E3 ligase activity, and the two MIU domains bind to ubiquitylated H2A. RNF168 functions in the monoubiquitination and K63 polyubiquitination of H2A/H2AX K13/15 that occurs after DSBs (Figure 1). RNF168 catalyzes H2A/H2AX monoubiquitination, whereas RNF8, a RING finger ubiquitin ligase, alone is insufficient to induce H2A/H2AX ubiquitination. RNF168 is responsible for the monoubiquitination of H2AK13/15, and RNF8 is efficient in extending its monoubiquitination to form a K63-linked ubiquitin chain [38]. RNF8 assembles at DSBs through the interaction of its FHA domain with the phosphorylated adaptor protein MDC1 at DSB and recruits RNF168 for K63 polyubiquitination and other downstream effectors [38,39,40,41]. There is extensive evidence on how RNF8 manages the recruitment of RNF168. It was first demonstrated that RNF8 and UBC13 (also known as UBE2N), an E2 ubiquitin-conjugating enzyme, mediate the K63-linked ubiquitinate H1-linker histone, promoting the recruitment of RNF168 that monoubiquitinates H2A at lysine 13/15 [42]. It was also demonstrated that ataxia telangiectasia mutated (ATM)-induced phosphorylation of L3MBTL2 induces the interaction of MDC1 at DSB, which is subsequently ubiquitylated by RNF8. Ubiquitylated L3MBTL2 facilitates RNF168 recruitment to the damage sites and promotes H2A polyubiquitylation [43]. These data suggest that RNF8-induced ubiquitination of more than one protein mediates the recruitment of RNF168 to the damage site [26,27].

**Figure 2:**
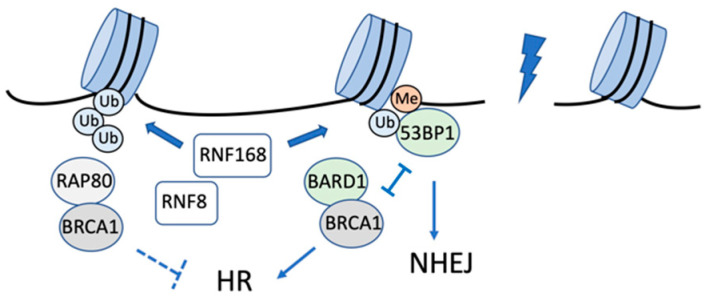
Model of H2AK13/15 ubiquitination and reader proteins in the DNA damage response. RNF168-induced H2AK13/15 ubiquitination leads to the activation of the NHEJ (53BP1) or HR (BRCA1/BARD1) pathway. RNF8-RNF168 induced K63-linked chains to recruit the BRCA1-A complex (RAP80/BRCA1) and restrict the HR pathway.

RNF168-induced monoubiquitination of H2AK15 is directly bound by the ubiquitin-dependent recruitment (UDR) motif of tumor protein 53 (TP53)-binding protein 1 (53BP1), which results in its accumulation at the DSB sites [44] (Figure 2). 53BP1 is a key regulator of NHEJ to inhibit DNA end resection, the initiation step of HR [30]. Furthermore, 53BP1 binds lysine 20 dimethylation on histone H4 (H4K20me2) through a tandem Tudor domain [45]. The two domains of 53BP1 are essential for its accumulation at the damage sites [30]. Alternatively, the NuA4/TIP60 (histone acetyltransferase KAT5) acetyltransferase complex also binds H4K20me2, and its binding competes for 53BP1 binding [46]. The Tip60 acetyltransferase complex is recruited to the DSB to acetylate H4, H2A, and H2AX, promoting chromatin relaxation [47,48,49]. In addition, Tip60-dependent H4 acetylation attenuates 53BP1 binding to H4K20me2 through the Tudor domain [8,24,50]. Moreover, the Tip60 complex can acetylate H2AK15 and its acetylation mutually exclusive ubiquitination by RNF168 [46]. Furthermore, Tip60 deficiency suppresses HR in a 53BP1-dependent manner. These results indicate that Tip60 competitively inhibits the recruitment of 53BP1 to chromatin, thereby suppressing 53BP1-dependent repair. Meanwhile, the tumor suppressor protein BRCA1-associated RING domain protein 1 (BARD1) interacts with the monoubiquitin of H2AK13/K15 through a BRCT domain UDR motif (BUDR) [31,32,33,34,35,51] (Figure 2). In addition to its binding to H2AK15ub, BARD1 interacts with lysine 20 of histone H4 in its unmethylated form (H4K20me0), but not with H4K20me2, through an ankyrin repeat domain [52]. Consequently, 53BP1 and BARD1–BRCA1 compete for the same damage-induced histone marker, and the BARD1–BRCA1 complex can specifically inhibit 53BP1 binding through dual recognition of H2AK15ub and H4K20me0. The RNF8/RNF168-induced K63 polyubiquitination of H2AK15 interacts with RAP80 throughits ubiquitin-interacting motif to promote the recruitment of the BRCA1-A complex. The BRCA1-A complex contains BRCA1/BARD1, RAP80, DUBs BRCC36, and BRCC45 [53,54]. RAP80 targets the complex to the site of DNA damage, where the complex limits DNA end resection to prevent excessive HR [22,55] (Figure 2).

Like other posttranslational modifications, such as phosphorylation, ubiquitination is reversible, and deubiquitination can be accomplished by specific DUBs to prevent excessive ubiquitylation. DUBs, including ubiquitin-specific proteases, ubiquitin C-terminal hydrolases, ovarian tumor proteases (OTUs), and Machado–Joseph Disease protease family (MJDs), are responsible for the cleavage of ubiquitin chains from substrate proteins [56]. Numerous DUBs have been demonstrated to be involved in the DNA damage response (Table 1). USP3 [57], USP16 [58], USP29 [59], USP44 [59], USP49 [60], USP51 [61], A20 [62], BRCC36 [63], and OTUB1 [64] have been suggested to inhibit DSB-induced histone H2A ubiquitination. Overexpression of USP3 and USP44 suppresses the localization of 53BP1 to the damage site. This suggests that these DUBs negatively regulate the recruitment of downstream factors after RNF8/RNF168-generated H2A ubiquitination [45,59]. The recruitment of USP44 to the damage site was initiated in an RNF8- or RNF168-dependent manner and therefore may facilitate the hydrolysis of ubiquitination induced using RNF8/RNF168 [59]. USP3-deficient mice exhibited short lifespan, increased tumorigenesis, and spontaneous genomic damage in MEFs [65]. USP49 overexpression suppressed ubiquitylation of γH2AX and DSB-induced foci formation of 53BP1. This resulted in hypersensitivity to DNA-damaging anticancer treatments [60]. Overexpression of USP51 suppresses the formation of ionizing radiation (IR)-induced 53BP1 and BRCA1 but not RNF168 foci, suggesting that USP51 functions downstream from RNF168 in the DNA damage response [61]. OTUB1 binds to UBC13 (UBE2N), an E2 ubiquitin-conjugating enzyme for RNF168, and restricts its interaction with RNF168, suppressing the DSB responses by inhibiting RNF168-induced polyubiquitylation independently of its catalytic activity [64]. Furthermore, USP11-mediated histone deubiquitination, together with NurD-mediated histone deacetylation, promotes the termination of the DNA damage repair response and results in the reconstruction of chromatin structure [66]. Further research is necessary to elucidate the function of these DUBs.

**Table 1:** Histone lysine ubiquitination enzymes and deubiquitination enzymes involved in DNA damage response.

Histone	Target	E3	DUB	Reader	Function	Refs.
H2A	K13/15	RNF8/RNF168	USP3, USP29 USP44, USP51,	53BP1, BARD1	Recruitment of 53BP1 or BARD1 to DSBs	[31,32,33,34,35,38,39,40,41,42,51,52,55,56,57,58,61,62,63,64,65,66]
	K127/129	BRCA1/BARD1	USP48	SMARCAD1	Recruitment of SMARCAD1 to promote end resection for HR	[35,67,68]
	K118/K119	RING1A/RING1B	BAP1, USP16	RYBP, JARID2, ZRF1	Transcriptional repression	[69,70,71,72,73,74,75,76,77,78,79]
H2B	K120	RNF20/RNF40	USP22, USP11	DOT1L	Transcriptional regulation, Recruitment of damage proteins	[12,80,80,81,82,83,84,85,86,87]
H2AX	K13/15	RNF8/RNF168	USP3, USP49,	53BP1	Recruitment of 53BP1 to DSBs	[38,41,60]
H3	K14/18/23	UHRF1	USP3, USP29 USP44, USP51,	DNMT1	Maintaining DNA methylation during DNA replication	[88,89,90]
		CUL4/DDB/ROC1	USP48	53BP1, BARD1	Recruitment XPC to the damaged foci after UV irradiation	[91]
H4	K91	BBAP	BAP1, USP16	SMARCAD1	Modulation of 53BP1 foci formation	[92]
		CUL4/DDB/ROC1	USP22, USP11	RYBP, JARID2, ZRF1	Recruitment XPC to the damaged foci after UV irradiation	[91]
H1		RNF8	USP3, USP29 USP44, USP51,		Recruitment of RNF168 to DSBs	[42]

### 2.2. H2AK127/129 Ubiquitination and Repair Pathway Choice

The tumor suppressor protein, breast and ovarian cancer predisposition protein-1 (BRCA1), promotes distinct steps of DSB repair by HR and protects DNA replication forks. Cancers originating from germline mutations in the BRCA1 gene cannot be repaired by HR and are sensitive to exogenous DNA-damaging agents such as PARP1 inhibitors (PARPi) or platinum [93]. BRCA1 counteracts the activity of the 53BP1–RIF1–Shieldin complex, protecting DSB ends from 5′-end resection, and activates the resection of DNA ends. DNA end resection is a critical step in the HR repair pathway. The BRCA1-induced resection of DSBs provides 3′ single-stranded DNA (ssDNA) overhangs and promotes RAD51 filament formation [94]. However, the precise molecular mechanism by which BRCA1 activates resection against the 53BP1 complex is unclear. BRCA1 may physically reposit 53BP1 from the DSB site [67,95] or recruit phosphatases that dephosphorylate 53BP1, resulting in the loss of binding between 53BP1 and RIF1 [96]. As BRCA1 is a component of several different protein complexes, it exerts its molecular function in HR owing to its interaction with proteins and the formation of different multiprotein complexes that are composed of different constituent factors and localize to the damage site in different ways [97]. The above-described formation of the BRCA1–RAP80 complex is governed by the interaction between RAP80 and RNF168/RNF8 catalyzed by K63-linked ubiquitin chains on chromatin surrounding the DSBs [53]. The BRCA1-A complex, including RAP80, was shown to restrict HR rather than promote DNA end resection during the S/G2 phase of the cell cycle [54,55]. It has been demonstrated that RAP80 binds ubiquitinated H2B following DNA damage; however, its significance in DNA repair remains unclear [98]. BRCA1 has several functional domains, including the RING domain, BRCT repeats, a coiled-coil (CC) domain, and a central unstructured region encoded by exon11. BRCA1 and BARD1 interact through their respective N-terminal RING domains. BRCA1 is recruited to the DSB sites through the interaction with BARD1 in the monoubiqitination of H2AK13/K15 [31,32,33,34,35,51]. BRCA1 and BARD1 form a heterodimer and exhibit E3 ubiquitin ligase activity due to the RING domain at the N-terminus of these proteins, and they may promote nucleolytic resection through its interaction with the CtBP-interacting protein (CtIP) in an MRN (RAD50/NBS1/MRE11)-dependent manner [97,99]. The BRCT repeat of BRCA1 forms a phosphopeptide-binding region that facilitates interaction with proteins such as CtIP, ABRAXAS, and BACH1. Recently, nucleosomal histone H2A has been identified as a substrate for the BRCA1–BARD1-dependent E3 ligase activity in DNA repair. The heterodimeric RING domains are sufficient for promoting the ubiquitylation of lysine residues 125, 127, and 129 of H2A, and also the ubiquitylation of lysine residue 123 of the histone variant macroH2A1 [35,68]. The ligase activity of BRCA1–BARD1 contributes to the function of BRCA1 in DNA end resection, which is required before the formation of the RAD51 nucleofilament in HR repair. 53BP1 and its effector protein, human REV7 or Artemis, suppress DNA resection in the absence of BRCA1 [100]. The recruitment of BRCA1 to the DSB sites is associated with 53BP1 removal from DSBs and initiates long-range DNA end resection and HR [95,101]. It was demonstrated that the ubiquitination of H2A K125/K127/K129 recruits the SWI/SNF-related, matrix-associated, actin-dependent regulator of chromatin, subfamily A, containing DEAD/H box1 (SMARCAD1) through its ubiquitin-binding CUE domains. SMARCAD1 was found to be required for chromatin remodeling, 53BP1 repositioning, DNA end resection, and HR [67]. BRCA1–BARD1-induced H2A ubiquitination and subsequent SMARCAD1-dependent 53BP1 repositioning are critical regulators of DNA repair [67]. These findings emphasize the multifaceted and complex function of BRCA1 in the regulation of DNA end resection and promotion of RAD51 loading, as well as the importance of BRCA1 in the pathway selection for DNA damage repair. BRCA1 also directly binds to PALB2 through their respective CC domains, forming a macrocomplex comprising BRCA1–PALB2–BRCA2–RAD51 that directs RAD51-loading onto ssDNA [94,102]. However, BRCA1-independent loading of PALB2 occurs through the activity of ATR and RNF168, and the importance of its interaction remains controversial [103,104,105,106]. USP48 has been identified as an H2A DUB, specific for the BARD1–BRCA1-induced ubiquitination of H2A K125/K127/K129 but not for H2AK119 or H2AK13/15 [107]. Overexpression of USP48 shortens the length of DNA end resection. However, USP48 loss increases DNA end resection and RAD51 foci formation as in the case of 53BP1 depletion, suggesting that USP48 antagonizes BRCA1 function and restricts RAD51 accumulation for promoting genome stability [107].

### 2.3. H2AK119 Monoubiquitination Promotes Transcriptional Regulation in the DSB Response

In the DNA damage response, histone H2A is ubiquitinated at different sites and thus plays different physiological roles. After the occurrence of DSBs, ubiquitylation at lysine 125/127/129 by BRCA1/BARD1 E3 ubiquitin ligases promotes HR repair [67]. Moreover, studies have reported that ubiquitination of lysine 13/15 by RNF168 is a possible basis for both the NHEJ and HR pathways [31,32,33,38]. H2A is also monoubiquitylated on lysine K119 (H2AK119ub1) [69], which induces transcription repression. This is promoted by the polycomb-group protein (PcG)-containing complex, especially polycomb repressive complex 1 (PRC1) [37,70]. The core component of the PRC1 complex, E3 ubiquitin ligase RING1A/RING1B, stimulates the monoubiquitination of H2AK119 [27,71,72,73]. H2AK119ub1 deposition is associated with repressed genes, particularly PcG-target genes, and colocalized with H3K27 trimethylation (H3K27me3), which is critical for transcription repression catalyzed by PcG repressive complex 2 (PRC2) [108,109,110]. After DNA damage, both the PRC1 and PRC2 subunits are recruited to the damage site, suggesting a role for H2AK119ub and H3K27me3 in transcription silencing in the damaged chromatin [111,112,113]. The repression of transcription in neighboring DSBs prevents conflict between transcription machineries and DNA repair [114,115,116]. Recent studies have suggested that BMI1 and RING1A/RING1B heterodimer facilitate DSB repair, which is dependent on their E3 ligase activity toward H2AK119; moreover, they contribute to γ-H2AX foci formation and recruitment of damage response factors [74,75]. In addition, BMI-mediated H2AK119 ubiquitylation induces DNA end resection, allowing the recruitment of downstream factors, such as replication protein A (RPA), BRCA1, and RAD51, at DNA damage sites, as well as the progression of HR. H2AK119ub could recruit the resection factor CtIP, interacting with ubiquitin moieties at the DSB site [36,76,112].

BAP1 is a deubiquitinase of the ubiquitin carboxyl-terminal of the hydrolase family that regulates gene expression and other cell functions through the deubiquitination of histone H2AK119ub [77]. It binds with ASXL1 to form the polycomb repressive deubiquitinase (PR-DUB) complex and deubiquitinates H2A [77]. Upon DNA damage, BAP is phosphorylated and recruited to the DSB sites in an ATM-dependent manner [78]. BAP1 deficiency inhibits efficient HR repair and increases radiation sensitivity, suggesting that the deubiquitination of H2AK119ub promotes HR [78]. Another DUB, USP16, also deubiquitinates H2AK119ub and promotes the reversal of transcription silencing [79].

### 2.4. H2B Ubiquitination and the DNA Damage Response

Ubiquitination of H2AK13/15 has been primarily implicated in the DNA damage response, and monoubiquitination of H2AK119 is involved in transcriptional activity silencing, whereas monoubiquitination of H2B promotes transcriptional elongation and activation. This monoubiquitination regulates replication, DNA damage response, cellular proliferation, and developmental plasticity [30,35,117,118]. In mammals, the E3 ligase RNF20/RNF40 heterodimer catalyzes the monoubiquitination of H2B at lysine 120 (H2B K120ub1) [80,81,82] and the deubiquitination modules of the SAGA complex, composed of ATXN7 (homolog of Sgf73), ATXN7L3 (homolog of Sgf11), ENY2 (homolog of Sus1), and USP22 deubiquitinate H2B [83,84]. Ubiquitination and deubiquitination of H2B are implicated in transcriptional regulation and DNA damage response [85,86]. The level of H2BK120ub1 increases following exposure to IR or treatment with radiomimetic drugs, suggesting the role of H2BK120ub in DSB repair [80,81,82]. Upon DNA damage, the ATM kinase phosphorylates the Ser172 and Ser553 residues of RNF20 and Ser114 of RNF40, facilitating the recruitment of the RNF20/RNF40 heterodimer to the DSB site and the catalysis of H2Bub1 [12,81,82]. Like the phosphorylated histone H2AX (γ-H2AX) by ATM, H2Bub1 accumulates in the vicinity of DSBs [82]. This reaction is not involved in the very early step of accumulation of damage sensor proteins, such as 53BP1 and MDC1; however, it is required for the timely recruitment of the NHEJ and HR factors, such as XRCC4, KU80, RPA, RAD51, RAP80, and BRCA1. In this reaction, H2BK120ub1 induces chromatin opening and serves as a platform for both the NHEJ and HR proteins, promoting optimal DSB repair through both pathways [80,81]. It has been reported that the depletion of the RNF20/RNF40 heterodimer resulted in the defects of class switch recombination (CSR), indicating that the heterodimer is required for the distal end joining of DSBs, i.e., efficient NHEJ [82]. It has also been reported that the RNF20/RNF40 heterodimer is required for DSB repair by HR. Altogether, RNF20 and RNF40 function in the DNA damage response proximal to a choice of either the NHEJ or HR pathway [28]. RNF20 is also required for the induction of the chromatin remodeling factor SNF2H to the DSB site, suggesting a role for H2BK120ub1 in chromatin remodeling at the DSB site [80,118].

H2B lysine 120 (H2BK120) is either acetylated or ubiquitinated. The conversion between the ubiquitination and acetylation of H2AK120 is performed by the PCAF and SAGA complexes [82]. USP22, a deubiquitinase of the SAGA complex, removes ubiquitin from H2BK120ub during the repair of programmed DSBs in B cells [87]. USP22-induced deubiquitination is critical for CSR, activation-induced cytidine deaminase, and IR-induced DSB repair, and the H2Bub level was found to be increased in USP22-deficient splenic B cells [87]. USP11 also deubiquitinates H2BK120 and H2AK119. USP11 has been demonstrated to be associated with the NuRD complex and related to efficient DNA repair, inducing chromatin condensation and genome stability [66].

### 2.5. Ubiquitination of H3 and H4 in the DNA Damage Response

Histones H3 and H4 are also ubiquitinated; however, their role in DNA damage response remains unclear. The well-characterized ubiquitination of H3 is H3K14/18/23 [88,89,90]. During DNA replication, the RING domain of ubiquitin-like with PHD and RING finger domain 1 (UHRF1) ubiquitinate histone H3 tail at K14/18/23, which are then recognized by DNMT1 to methylate hemimethylated DNA. DNA interstrand crosslinking (ICL) is a major type of DNA damage during DNA replication, and the FA pathway is mainly responsible for ICL repair. The major function of the FA pathway is presumably the regulation of SLX4/FANCP, the ICL lesion-processing nuclease, by the monoubiquitination of FANCD2/FANCI [119,120,121,122,123]. Intriguingly, UHRF1 functions in ICL repair by binding to ICL lesions that are FA pathway-independent barriers to DNA replication and recruiting ICL repair nucleases [9]. Hence, UHRF1 functions not only in maintaining DNA methylation but also in the DNA damage response. Furthermore, a ubiquitin ligase complex, comprising CUL4A, CUL4B, DDB1, DDB2, and ROC1 (RBX1), catalyzes H3 and H4 ubiquitylation in response to UV irradiation [91]. In addition to these H3 or H4 modifications [91], H4K91 ubiquitination has been found to occur after DNA damage [92], although its detailed mechanism requires further investigation.

## 3. Conclusions

Eukaryotic cells respond to various genotoxic stresses, and histones and their variants proximal or distal to the DNA damage site undergo posttranslational modifications to regulate chromatin structure. Histone modifications are closely associated with the initiation, progression, and convergence of the DNA damage repair response. The function of histone modifications in DNA damage repair depends on reader proteins that directly bind to modified residues (Table 1). Histone methylation readers recognize methylated residues through several different domains, such as chromodomain (CD), malignant brain tumor (MBT), Tudor, and plant homeodomain (PHD). Similarly, there are several proteins with ubiquitin-binding domains that are reader proteins and recognize histone ubiquitination following DNA damage. Several reader proteins recognize the ubiquitination of chromatin at several different sites, and the antagonistic recognition of ubiquitination of the same chromatin by different reader proteins also appears to select the optimal repair pathway for the damage [22,23]. Histone modification in the DNA damage repair reaction is also recognized by several proteins, among which the best-known is 53BP1, a central protein in the NHEJ pathway. As reviewed in this article, 53BP1 binds to H2AK15ub through a UDR motif and transfers it to the DSB site [30,44]. 53BP1 also directly binds to H4K20me2 in the tandem Tudor domain [45]. Contrarily, BARD1 binds to H2AK15ub through BUDR. BARD1 does not bind to H4K20me2 but binds to unmethylated H4K20me0 [31,32,51]. BARD1 also binds to BRCA1 and performs H2A ubiquitylation (K125/127/129), recruits SMARCAD1 to the DSB sites, and reassociates DNA ends by 53BP1 repositioning [67]. Therefore, BARD1 plays a central role in the HR pathway by binding to BRCA1 and antagonizing NHEJ in the repair response, indicating that the two reader proteins antagonistically recognize a common histone modification [67].

Histone modification has been shown to play a vital role in the selection of DSB repair pathways; however, it also plays an essential role in the interaction with transcription and replication. Ubiquitination of histone lysine residues regulates not only the DNA damage response but also various biological phenomena, such as DNA methylation maintenance and gene expression regulation. During DNA replication, UHRF1 catalyzes multiple monoubiquitination of H3 tail, which is then recognized by DNMT1 to methylate hemimethylated DNA [88,89,90]. Moreover, the regulation of transcription is closely related to DNA damage repair. Upon DNA damage, the chromatin proximal to the damage site is regulated by transcriptional repression to prevent conflict between transcription and repair [111,112,113]. Of these regulatory mechanisms, PRC1 and PRC2 are well known to be recruited to the damage site and induce H2AK119ub and H3K27me3, respectively [124]. Consequently, transcription is repressed in the chromatin proximal to the damage site, and binding to H2AK119ub recruits the DNA end resection factor CtIP to promote HR repair [76]. Therefore, histone modification, which functions in the DNA damage repair response, is also associated with transcription and replication. Nevertheless, only a few DNA damage response proteins have been shown to bind to histone modification. Further biochemical and structural analyses would demonstrate that many more proteins are histone modification readers. In addition, more detailed characterization of the chromatin structure at the damage site will provide a chronological understanding of the DNA damage response.
